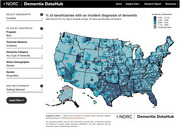# Introduction of an open‐access national‐level U.S. dementia surveillance system

**DOI:** 10.1002/alz70860_100503

**Published:** 2025-12-23

**Authors:** Melinda C Power, Kan Z Gianattasio, Wachsmuth Jason, Ryan Murphy, Alex Hartzman, Jaleh Montazer, Erin Cutroneo, John Wittenborn, David B Rein

**Affiliations:** ^1^ The George Washington University, Washington, DC, USA; ^2^ NORC, Bethesda, MD, USA

## Abstract

**Background:**

Surveillance is critical to public health planning, resource allocation, innovation in development of care models and treatments, and tracking of progress towards addressing efforts in prevention and disparity reduction. No federal or private system currently tracks and provides surveillance for Alzheimer's disease and related dementias (ADRD) in the U.S.

**Method:**

Our team used the 100% Medicare fee for service (FFS) and Medicare Advantage (MA) claims data develop a web‐based interface for national‐level U.S. surveillance of diagnosed dementia.

**Result:**

The Dementia Datahub (https://dementiadatahub.org/) currently provides mapping, data reporting dashboards, system documentation, and public use files (available upon request) of statistics from the year 2020. Available statistics include prevalence and incidence of diagnosed dementia, as well as mortality and COVID‐19 infections among prevalent dementia cases and all average all cause expenditures among prevalent dementia cases in fee‐for‐service Medicare. All estimates are available at the national, state, and county level, overall and within subgroups defined by age group, sex, race/ethnicity, and FFS/MA enrollment status. The tool additionally allows for bi‐variate analyses and overlay with state or county‐level measures of demographics, poverty and education levels, vision or hearing impairment, number of physicians per capita, and FFS/MA enrollment rates. This year we will add 2021 data and additional outcome measures of hospitalizations and long stay nursing home placement among prevalent dementia cases. If funding can be obtained, goals include expansion to add additional years of data, additional capabilities, and updating closer to real‐time.

**Conclusion:**

The Dementia DataHub provides an important resource to government, academic, industry, advocacy, and medical organizations working to address the epidemic of cognitive impairment and dementia.